# Experiences of Spouses of Women with Breast Cancer: A Content Analysis

**DOI:** 10.31557/APJCP.2019.20.10.3167

**Published:** 2019

**Authors:** Zeinab Younes Barani, Mozhgan Rahnama, Mahin Naderifar, Mahin Badakhsh, Hajar Noorisanchooli

**Affiliations:** 1 *Student Research Committee,*; 3 *Department of Nursing, *; 4 *Lecturer, Midwifery Department, Faculty of Nursing and Midwifery,*; 2 *Faculty of Nursing and Midwifery, Zabol University of Medical Sciences, Zabol, Iran. *

**Keywords:** Spouses, women with breast cancer, experiences, content analysis

## Abstract

**Introduction:**

In addition to the affected person, diagnosis and treatment of breast cancer also severely affects her husband. Therefore, it is worth paying attention to the needs of husbands of women with breast cancer. Therefore, the aim of the present study was to explain the experiences of spouses of women with breast cancer.

**Method:**

The present study was a qualitative study with conventional content analysis approach. Purposive sampling was carried out by selecting 6 spouses of women with breast cancer. Data were collected through semi-structured interview. The recorded interviews were transcribed verbatim. Content analysis was used to reduce and name the data, obtain analytical codes, and finally recognize the theme.

**Results:**

Data analysis resulted in the extraction of 4 categories of couples’ mental challenges, multifaceted romantic meditation, multifaceted traumas caused by the disease, dual energies (inductions) of relatives, and 12 subcategories.

**Conclusion:**

In spite of suffering from all the challenges and traumas, husbands of women with breast cancer have not left their wives alone and have done their best to improve their lives; so, we can raise ““*Scarifying your life to save your wife’s life*”” as an extract from the experience of spouses of women with breast cancer. Knowing and understanding this point by clinical staffs and policy makers can provide pave the way for planning to provide comprehensive support to these men.

## Introduction

Husbands make up 30-40% of informal caregivers in chronic diseases (Duggleby et al., 2012). Breast cancer also has direct effects on the husbands of these patients (Yusoff et al., 2012). Because breast cancer is not only a female breast disease, but also a disease involving both couples (Zahlis and Lewis, 2010). Making a good decision is an basic and important aspect in different situations (Jahanpour et al., 2010). As women experience cancer, their husbands often become fragile, they consider cancer a factor threatening their sexual partner’s life and fear that they will not be able to support and provide proper care for their wives (Neris and Anjos, 2014). Basically, evidence indicate high levels of psychological distress experienced by family caregivers, especially spouses of cancer patients (Li et al., 2013; Khalili et al., 2016); in other words, complication of cancer and its treatment, in addition to the patient, negatively affect the quality of life of their spouses who act as informal caregivers and provide the highest level of support for married women (Wagner et al., 2006). Basically, breast cancer-related needs of husbands of women with this disease are worthy of attention because if they are not helped, the disease and its subsequent pressure imposed on husbands can have detrimental consequences on the quality of couples’ relationship (Fletcher et al., 2010) in particular when these patients, in addition to psychological problems and disorders, also have problems with marital satisfaction and experience fragile relationship with their spouses (Shareh, 2016). Because the disease targets one of the most important sexual organs of women and is directly related to the sexual identity of women, and this can lead to impaired sexual and marital relationships (Harirchi et al., 2012); while these women expect mental support from their husbands (Çömez and Karayurt, 2014). Considering that the spouses of these patients can play a special role as a source of support for them (Wagner et al., 2006), on the one hand, and that successful management of patients and their family caregivers requires a thorough understanding of the experiences of those involved (Elahei et al., 2014); on the other hand, the aim of the present study was to explain the experiences of spouses of women with breast cancer using content analysis method. It is hoped that this study can provide the treatment team with a guide for implementing healthcare strategies by providing a clear picture of human experiences.

## Materials and Methods

The experiences of 6 spouses of women with breast cancer were evaluated in this qualitative content analysis study using a conventional approach. Participants were selected through purposive sampling. Data collection was carried out by the researcher by referring to the chemistry clinic of the hospital and the participants’ home after coordinating with them and obtaining their permission. First, the aim of the research was explained to them, and then the sampling was continued until data saturation. The criterion for achieving data saturation included the lack of achieving new concepts and codes in subsequent interviews.

The data collection method included semi-structured in-depth interviews using open-ended questions, including, “*Tell us about your experiences after knowing about your spouse having breast cancer? How has your life changed since your spouse developed breast cancer?*” During the interview, the researcher helped participants state their experiences without directing their conversations. Probing questions were also used if necessary. Face to face Interviews were conducted using audio recorder(Samsung) in a quiet environment and each interview lasted between 45-60 minutes in one or two sessions depending on the condition and patience of the participants. All interviews were analyzed by the recorded researcher, typed verbatim, reviewed, coded, and analyzed immediately. Two of interviews repeated by researchers. There was no dropout of participants and all of them participated. In fact, data analysis was performed simultaneously and continuously with data collection. Maxqda version 12 used for managing the data. Data were analyzed using conventional content analysis approach in a way that each interview was first read carefully for obtaining an initial grasp, important statements were underlined, and recorded as codes (initial coding). The participants’ own words and implicating codes (researcher’s perceptions of the statements) were used for initial coding. Then, codes that were conceptually similar to each other were modified and subdivided into categories and subcategories to clarify meaning. The data analysis process was performed according to the steps of Graneheim and Lundman (Graneheim and Lundman, 2004). In order to meet credibility, the coded interviews were given to the participants to confirm that they match with their experiences, and corrections were made in some cases. In order to meet the trustworthiness criterion, the codes and concepts obtained were consulted with and investigated by the experts and colleagues of the research project. Moreover, several colleagues were asked to decode some parts of the transcript of the interviews and then review the degree of agreement for coding. To confirm the transferability of the findings, attempts were made to select participants with different demographic characteristics and different experiences and the researcher assessed all aspects of behavior, events, and living experiences. The confirmability of findings was met by the detail-rich descriptions of all stages of the research, while the details of the research were carefully documented to allow the evaluation of external observers. The following ethical consideration were also taken into account: obtaining an introduction letter, obtaining informed consent from the participants for recording interviews, confidentiality of information, and allowing them to withdraw from the study.

## Results

The individual characteristics of the participants are presented in Table 1. The data analysis resulted in the extraction of 4 categories and 12 subcategories (Table 2).

*Couples’ mental challenges*

The experiences of spouses of women with breast cancer show that women and their spouses have experienced numerous mental challenges following the disease.

*Wife’s mental challenges*

According to the experiences of the spouses of women with breast cancer, these women were shocked after being informed about the diagnosis, were mentally disturbed, fearful of chemotherapy, feeling depressed, indifferent towards themselves, crying, and brooding in a way that preferred solitude, isolation, and staying at home. They also felt alienation with their spouse and showed some sort of emotional withdrawal with him so that they avoided verbal communication with him and felt that they could no longer be a women of married life. As one of the participants said about his wife’s isolation:

*“My wife has been senseless since she become ill and stays in a corner, she is lethargic due to the medications she is taking, she does not even talk to me and children.” (P. 4)*

*Husband’s mental challenges*

According to the experiences of the spouses of women with breast cancer, after being informed about their wife disease, these men were shocked and denied it, were psychologically disturbed, were upset with the changes in their spouse’s appearance, felt depressed, and got bored of messy situation in their life, had permanent mental conflicts due to problems caused by their spouse’s disease, feared the spread of the disease and the possible death of their spouse, had lower tolerance threshold, and became aggressive. In this regard, one participant reported his shock after being informed about his wife’s disease:

*“It was shocked when the doctor said that my wife had breast cancer. It was hard to believe and I said oh, my God what was this? (P. 2)*

*Multifaceted romantic meditation*

The experiences of the spouses of women with breast cancer showed that these men were meditating on their wives in mental, therapeutic, sexual, and spiritual aspects because they loved their wives very much.

*Romantic mental meditation*

According to the experiences of the spouses of women with breast cancer, these men had made every effort to maintain the morale of their spouse, didn’t leave her alone, didn’t show sensitivity to the facial changes caused by the disease, gave no sign of disease problems, and behaved normally so that their spouses would not be embarrassed by these changes, consoled their wife, and said that they still loved her and were not thinking of remarriage. To avoid pity, they have restricted relationship with relatives or hid the disease. They were trying to help the patient get rid of mental obsession and remove them from isolation by telling jokes. In this regard, one of the participants stated about his attempt to maintain his wife’s morale:

*“I stopped mixing with my coworkers and neighbors because of my wife’s disease because I don’t want them to ruin my wife’s morale with their words.” (P. 1)*

*Therapeutic-romantic meditation*

According to the experiences of the spouses of women with breast cancer, these men did their best for the treatment of their spouses to the extent they accompanied her during treatment, paid for medical care costs, and took care of her after chemotherapy. In this regard, one of the participants stated about his effort to cover the cost of his spouse’s treatment:

*“Even though I’m a worker, but I swear to God that I did not fail to treat my wife, I even borrowed(money), but paid for her medications. If I spend money much higher for my wife, it is still low.” (P. 5)*

*Sexual-romantic meditation*

According to the experiences of the spouses of women with breast cancer, to observe their wives’ conditions, these men had reduced their sexual intercourse with their spouse considering their spouse’s reduced sexual desire and pain during the intercourse. In this regard, one of the participants described their sexual intercourse as follows:

*“Because of the chemotherapy drugs, my wife doesn’t want to have sex, so I showed my consideration to her conditions and I don’t ask for sex so much.” (P. 3)*

*Spiritual-romantic meditation*

According to the experiences of the spouses of women with breast cancer, these men sought to mediating their spouse by turning to spirituality through relying on God, vowing, appealing to the Ahlul-Bayt, and increasing their spiritual tendencies. As one of the participants said in this regard:

*“When I found out that my wife was having cancer, I told her it must have been the God’ will and we started treating her with trust in God.” (P. 2)*

*Multifaceted traumas of the disease*

The experiences of spouses of women with breast cancer showed that these men were traumatized in different respects by their spouse’s disease.

*Trauma to role playing*

According to the experiences of spouses of women with breast cancer, these men have had problems with playing their role in the workplace due to their caregiving role, such as absenteeism or use of unpaid leave due to lack of paid leave and fatigue. In addition to taking care of the patient and assuming their job duties, they also had to endure the double burden of household chores to help their spouse with their household chores since they face with their spouse’s disability. As one of the participants stated about unwanted absences from work due to his spouse’s disease:

*“While I was involved with my wife’s disease, I was frequently absent from work. My job task could remain unfinished.” (P. 4)*

*Financial damage*

According to the experiences of the spouses of women with breast cancer, these men had financial difficulties in paying for medical care costs so that they experienced imbalances in income and costs and sometimes had to discontinue chemotherapy due to their inability to afford the costs. As one of the participants described her inability to pay for his spouse’s treatment costs as follows:

*“The doctor told recommended chemotherapy for my wife, where do I get money to pay one million seven hundred thousand tomans for chemotherapy? They told me to have six chemotherapy sessions for my wife but I don’t have the money for one session, let alone pay for 6sessions”. (P. 6)*

*Traumas to married life*

According to the experiences of the spouses of women with breast cancer, these men had experienced numerous traumas to their married lives, including convulsions, cold and shortness of breath, disruption of the normal routine, and disintegration of the married life. As one of the participants said about the feeling of disintegration of their married life after their spouse got cancer:

*“After this disease, it seems as if our lives were disintegrated, we were no longer living as before, and we were only thinking about the treatment of my wife.” (P. 3)*

*Traumas to children*

According to the experiences of the spouses of women with breast cancer, these men had experienced damage to their children’s body following the disease, including crying, grief, fear, shock, worry, and educational failure. As one of the participants described children cried due to their mother’s disease:

*“My spouse’s hair and eyebrows fell out during chemotherapy. My kids started crying when they saw their mother in this condition and told her what we would do without you if you died? If you die we would have no mom.” (P. 5)*

*The dual energies (inductions) of relatives*

The experiences of spouses of women with breast cancer showed that these men are positively and negatively influenced by two types of energy (inductions) of the relatives.

*Positive energy (inductions) of relatives*

According to the experiences of the spouses of women with breast cancer, these men felt positive energy through empathy, financial support, and psychological support from relatives. If one of the participants explained family financial support as follows:

*“Our family has been very supportive of us, even though they live in the village and have no income, they still sell their sheep, which were all they have and gave us the money to spend on treatment.” (P. 3)*

Negative energy (inductions) of relatives According to the experiences of the spouses of women with breast cancer, some of relatives bothered these men by inducing the futility of treatment, incorrect (disabilities) restrictions, and feeling guilty, showing pity, and sympathy for the patient, and encouraging the men to remarry.

*“Since my wife has had breast cancer, my family has put mw under pressure several times to leave my wife and marry another woman.” (P. 4)*

## Discussion

*Couples’ mental challenges*

In the present study, husbands of women with breast cancer pointed to the mental challenges of their wives. Joulaeeet al., (2012) study, women with breast cancer reported experiences of living with cancer as losing important things, lack of self-confidence, living with fear, psychological dysregulation, and feeling the need to supported in the face of the negative aspects of living with cancer. However, Khansari et al., (2016) concluded in their study that although most breast cancer patients had feelings such as anger and sadness at the time of diagnosis, but they get along with the disease and its complications over time. They have got along with this issue and none of them regarded this change as a negative experience. This result is somewhat different from the result obtained in the present study and such difference may be due to the fact that Khansari et al., (2016)’s study focused on the psychological experiences of women with breast cancer and the present study identifies the psychological experiences of these patients indirectly from their husbands’ comments.

**Table 1:** Individual Characteristics of Spouses of Women with Breast Cancer

Participant	Age	Job	Level of education	Years of living together	Duration of the patient's breast cancer or duration of patient care	Number of children
P.1	44	Manual worker	Second-grade secondary school	19 years	1 year and 5 months	2
P.2	32	Employee	Bachelor	5 years	3 years	-
P.3	40	Employee	Associate Degree	10 years	4 years	2
P.4	47	Mason	Cycle	26 years	10 months	3
P.5	37	Self-employed	Diploma	12 years	1 year	-
P.6	31	Teacher	Bachelor	4 years	2 years	1

**Table 2:** Main Categories, Extracted Categories and Subcategories

Main Category (Final Theme)	Categories	Subcategories
Scarifying your life to save your wife’s life	Bilateral mental challenges	Women's mental challenges Husband's mental challenges
	Multifaceted romantic meditation	Romantic mental meditation Romantic therapeutic meditation Romantic sexual meditation Romantic mental Meditation
	Multifaceted traumas of the disease	Trauma to role playing Financial trauma Causing trauma to the married life Causing trauma to children
	The dual energies(inductions) of relatives	Positive energy (inductions) of relatives Negative energy (inductions) of relatives

In the present study, husbands of women with breast cancer pointed to a number of mental challenges. Duggleby et al., (2012) and Lopez et al., (2018) also referred in their studies to the exposure to psychological distress as experiences of husbands with breast cancer. Alacacioglu et al., (2014) concluded that sexual partners of cancer patients are affected by psycho-social and psycho-sexual effects of cancer more greatly than cancer patients. Also Bigatti et al., (2011) showed in a study that spouses of cancer patients had a high depression score.

*Multifaceted romantic meditation*

The experiences of the spouses of women with breast cancer showed that these men were meditating on their wives through their spiritual, therapeutic, sexual, and spiritual aspects considering their great love for their spouses. The results of a qualitative study by Lin et al., (2013) also showed that husbands of women with breast cancer, despite facing great suffering, strived to live a better life with their wives, to fulfill their responsibilities, and to maintain the love between themselves and their spouse. The results of the Youssef et al., (2012) and Mentford et al., (2016) studies also showed that husbands of women with breast cancer had a supportive role for their wives. With regard to the romantic spiritual meditation, husbands of women with breast cancer supported them by focusing on their women’s feelings and providing appropriate relevant care (Zierkiewicz and Mazurek, 2015). Duggleby et al., (2012) also found that husbands of women with breast cancer mentally supported their wives. With regard to therapeutic romantic meditation, husbands of breast cancer women participated throughout their women’s healing process (Zierkiewicz and Mazurek, 2015). With regard to the romantic-sexual meditation and spiritual-romantic meditation, mental challenges were also one of the themes obtained in a qualitative study carried out by Youssef et al., (2012) who examined experiences of husbands with breast cancer, because these women induction they were no longer attractive to their husbands and had negative inductions in this regard. Another theme was psychological defense, pointing out that these prevent mental problems by using spiritual affairs. However, in a study titled “*Experiences of women with breast cancer in relation to their spouses,*” Sanchooli et al., (2017) showed that all of them had no similar experiences. Some women had unpleasant experiences, such as rejection and helplessness, and felt that they were oppressed by their husbands, were unwilling to continue their married life, and had somehow felt being imposed. However, some of them had pleasant experiences and were satisfied with their spouse’s spiritual companionship, spiritual meditation, sexual meditation, and cooperation during their treatment. Such discrepancy may be somewhat attributed to the study population.

*Multifaceted traumas of the disease*

The experiences of the spouses of women with breast cancer showed that these men were affected by their spouse’s disease in various aspects, including role playing, financial, married life, and children. To confirm the trauma to role playing, Montford et al., (2016) showed in a study that some experiences of spouses of women with breast cancer included their altered roles and participation in household chores. Husbands of women with breast cancer also managed all household chores throughout the treatment period (Zierkiewicz and Mazurek, 2015). Alacacioglu et al., (2014) also writes that spouses of cancer patients face numerous problems, including work-related and lifestyle distress, and poor quality of life. They are required to support their spouses in daily activities and to assume greater responsibility for household chores and children. To confirm the financial trauma, Lopez et al., (2018) showed in their review study that they posed economic problems as experiences of husbands of women with breast cancer. To confirm trauma to married life, the results of Khademi and Sajadi, (2009) ’s study also showed that patients with breast cancer and those involved in the disease experienced a disturbance in their daily activities. To confirm the trauma to children, Mazzotti et al., (2012) also concluded that cancer is a real threat to the stability of the maternal role since it reduces their ability to care for their children. Möller et al., (2014) also concluded that, their children experience more emotional and behavioral problems in response to this diagnosis due to their mother’s disease. Kim et al., (2012) also concluded that one of the concerns of mothers with breast cancer is the permanent challenges their caregiving role because they have to pay attention to themselves due to the disease, but such attentions act as an interfering factor in relation to their children. To confirm this finding, Vaziri et al., (2014) writes that breast cancer diagnosis and treatment necessitates new family roles, as maternal focus is on treatment, and the disease interferes with maternal role and performance, relationship with children, and children performance.

*The dual energies (inductions) of relatives*

The experiences of spouses of women with breast cancer showed that these men are positively and negatively influenced by two types of energy (inductions) of the relatives. To confirm the positive energy (induction) of the relatives, Esmaeili et al., (2012) referred to support from family and relatives as one of the categories of data extracted. LeSeure and Chongkham-ang, (2015) also writes that family caregivers play an important role in supporting the patients and such care is a full-time job for them. To conform the negative energy (inductions) of relatives, the results of Sanchooli et al., (2017)’s study also showed that some of these women received negative feedback from their spouse’s family in the form of stimulating their spouses for separation and despair and consequently they had experienced a sense of helplessness. Probably one of the reasons for negative energy inductions from relatives is their lack of awareness or inability to adapt to existing conditions and solve existing problems. Consistent with this finding, Sajjadian et al., concluded in their study that sufficient attention must be paid to the patient care to provide better quality care to patients. The greatest need of caregivers seems to the lack of specialized information on breast cancer and provision of care to themselves and the patient (Hydary and Mokhtari Hesari, 2015). Micharoen et al., (2013) also write that family caregivers are vulnerable and at-risk population ignored by healthcare providers and have not received the necessary attention because caregivers primarily focus on patients.

In conclusion, husbands of women with breast cancer despite all the challenges and traumas, they did not leave their wives alone with their disease and had done their best to help their wife recover. Therefore, we can pose “*Scarifying your life to save your wife’s life*” as the extract from the experience of spouses of women with breast cancer. Knowing and understanding this point by clinical staffs and policy makers can provide the basis for planning to provide comprehensive support to these men.
